# Supplementary material for: Splice-Junction-Based Mapping of Alternative Isoforms in the Human Proteome
Source: Cell Rep. Author manuscript; Available in PMC 2020 Jan 15. (PMC6961840; doi:10.1016/j.celrep.2019.11.026)

A

sp|Q16563|SYPL1_HUMAN|ENSG00000008282|SE1|43810|chr7|106091939|106092669|−2|r134|T4,sp|Q16563|SYPL
ETSLHSPSNTSAPHSQGGIPPPTGL q value: 8.8873e−05 Tr_novel:TRUE RefSeq_Novel:TRUE
Search result spec prec mz: 824.0733 Actual spec prec mz: 824.0733
Fragments matched per AA: 1.32 Proportion of top 20 peaks matched: 0.45

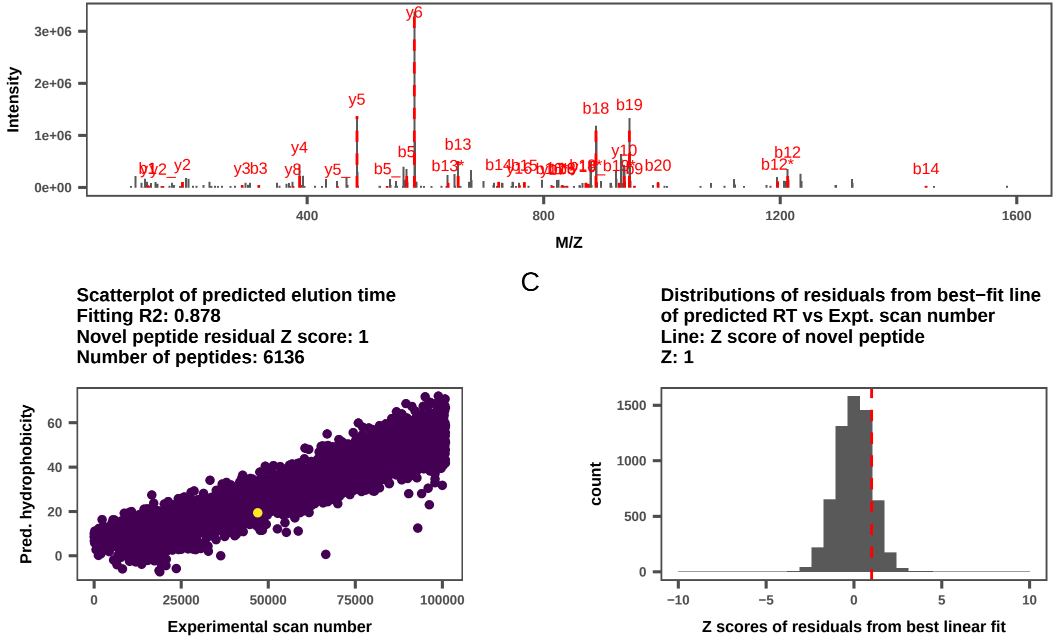

Supplement: 2 [file NIHMS1546469-supplement-2.zip › DF1/PXD006675/SmoothMuscleCells/SmoothMuscleCells_2_SYPL1_ETSLHSPSNTSAPHSQGGIPPPTGL.pdf]
